# Amplicon-based skin microbiome profiles collected by tape stripping with different adhesive film dressings: a comparative study

**DOI:** 10.1186/s12866-021-02122-4

**Published:** 2021-02-18

**Authors:** Kazuhiro Ogai, Kana Shibata, Natsuki Takahashi, Kohei Ogura, Shigefumi Okamoto, Junko Sugama

**Affiliations:** 1 AI Hospital/Macro Signal Dynamics Research and Development Center, Institute of Medical, Pharmaceutical and Health Sciences, Kanazawa University, 5-11-80 Kodatsuno, Kanazawa, Ishikawa 9200942 Japan; 2 Department of Nursing, Faculty of Health Sciences, Institute of Medical, Pharmaceutical and Health Sciences, Kanazawa University, 5-11-80 Kodatsuno, Kanazawa, Ishikawa 9200942 Japan; 3 Department of Clinical Laboratory Science, Faculty of Health Sciences, Institute of Medical, Pharmaceutical and Health Sciences, Kanazawa University, 5-11-80 Kodatsuno, Kanazawa, Ishikawa 9200942 Japan; 4 Advanced Health Care Science Research Unit, Institute for Frontier Science Initiative, Kanazawa University, 5-11-80 Kodatsuno, Kanazawa, Ishikawa 9200942 Japan

**Keywords:** Skin, Microbiome, Tape-stripping, Adhesive, 16S rRNA, Next generation sequencing

## Abstract

**Background:**

Medical film dressings have been used to obtain skin microbiota for skin microbiome studies, although their adhesive force may be so strong that the skin could be injured when applied to those who have fragile skin, such as older people. Several products with less adhesive force are available, although their applicability for skin microbiome studies remains unknown. This study aimed to test whether the dressings with less adhesive force could be used for amplicon-based skin microbiome studies. A set of three different film dressings, with acrylic, urethane, or silicone adhesive, was applied to the back skin of nine healthy young participants. The copy number of the 16S ribosomal RNA (rRNA) gene, microbial compositions, and alpha and beta diversity indices were analyzed by amplicon analysis of the 16S rRNA gene using next-generation sequencing and were compared among the three film dressings.

**Results:**

The dressing with acrylic adhesive yielded the highest copy number of 16S rRNA genes, followed by that with urethane adhesive. The silicone-adhesive dressing yielded a significantly lower copy number of the 16S rRNA gene. The microbial composition of skin microbiota was similar among the three film dressings, although significant differences in the relative abundance of *Pseudomonas* species and alpha diversity indices were found in the silicone-adhesive dressing. The Bray–Curtis dissimilarity was significantly higher between the acrylic- and silicone-adhesive dressings than between the acrylic- and urethane-adhesive dressings. No adverse effects related to tape stripping were observed for any of the film dressings.

**Conclusion:**

We recommend dressings with acrylic or urethane adhesive for amplicon-based skin microbiome studies. An acrylic adhesive has an advantage in the yield of skin microbiota, and a urethane adhesive should be chosen when applied to fragile skin. The adhesive force of the dressing with silicone adhesive was too weak to be used for collecting skin microbiota.

**Supplementary Information:**

The online version contains supplementary material available at 10.1186/s12866-021-02122-4.

## Background

As the largest organ, the skin serves as the physical barrier of our body, not only by protecting from external insults but also by retaining water inside the body [1]. Skin immunity also forms a barrier against the invasion of pathogenic bacteria or foreign substances [2]. In contrast, systemic diseases, such as diabetes, skin diseases, such as atopic dermatitis and psoriasis, or merely aging can affect the skin’s integrity, leading to a barrier breakdown [3].

The relationship between host and skin bacteria has long been discussed in terms of pathogenicity [4], immunological interaction [5, 6], and physiological barrier function [7]. Studies on cutaneous bacteria and its relationship with the host have been accelerated by the development of high-throughput sequencing techniques, namely, next-generation sequencing (NGS) analysis of the 16S ribosomal RNA (rRNA) [8, 9]. However, as the skin harbors more diverse and less abundant microbiota than the other parts of our body (e.g., gut), a more careful design of skin microbiome studies is required [10].

The factors that affect the results of skin microbiome studies include the method used to obtain the samples of skin bacteria (e.g., skin biopsy, swabbing, skin scraping, cup scrubbing, or tape stripping), the DNA extraction method, the region of the 16S rRNA gene to be sequenced, and the software and database employed [10]. Of them, the sampling method needs to be determined before performing a series of studies, as this cannot be changed after sampling.

Although the swabbing method has been widely used to collect skin microbiota for microbiome studies, the tape-stripping method has also been used due to its stability of collection maneuver, better microbiome coverage, and ease of use [11–13]. As skin microbiota exists across the layers of the stratum corneum, and the microbial composition can be different according to its depth [14, 15], the tape-stripping method is also preferred when focusing on the inner microbiome in the stratum corneum.

Several skin microbiome studies have successfully used medical-grade film dressings (thin, sterile, air-permeable medical tapes) for the tape-stripping method because they have been validated to obtain comparable results with traditional swabbing methods for collecting skin microbiome [12, 13]. However, one problem that is encountered with the use of film dressings is that its stickiness may cause skin injuries, especially when sampling from infants or older people who have fragile skin. The skin of older people is very thin, making it extremely fragile against even minimal invasion, including peeling off the tape [16–18]. Even with medical-grade film dressings, skin injuries may occur, called medical adhesive-related skin injuries (MARSI) [19]. To prevent MARSI, film dressings with less sticky adhesives than commonly-used acrylic adhesive, such as a silicone or urethane gel glue, have been introduced in clinical settings [16, 17, 20, 21]. When the tape-stripping method is used for skin microbiome analysis on those who have fragile skin, researchers need to choose less invasive adhesives, such as urethane or silicone adhesive, without sacrificing the yield and fidelity to the composition of the skin microbiome. Therefore, we first need to clarify whether there are differences in skin microbiome results when different adhesives are used.

The aim of this study was to compare the microbiome data obtained from three different film dressings with different adhesives (acrylic, urethane, or silicone) to test the applicability of these film dressings for amplicon-based skin microbiome studies.

## Results

### Adverse effects of tape stripping

No adverse effects during tape stripping (e.g., pain, redness, irritation, skin breakdown, etc.) were observed throughout this study.

### 16S rRNA gene copy number

**Fig. 1:**
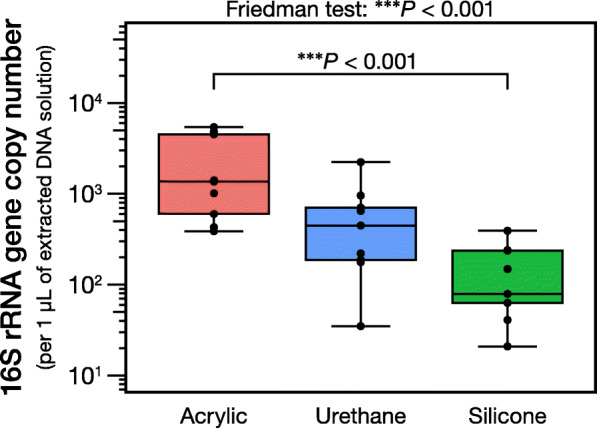
Copy numbers of the 16S ribosomal RNA (rRNA) gene collected by the three different film dressings. The vertical axis is a base-10 log scale. ^***^P < 0.001

Figure 1 shows the copy number of the 16S rRNA genes of each type of film dressing. The film dressing with silicone adhesive yielded significantly fewer copies of the 16S rRNA gene than that with acrylic adhesive (*P* < 0.001). There were no significant differences in the copy number of the 16S rRNA gene between the acrylic and urethane adhesives (*P* = 0.086) and between the urethane and silicone adhesives (*P* = 0.086).

### Bacterial composition

**Fig. 2:**
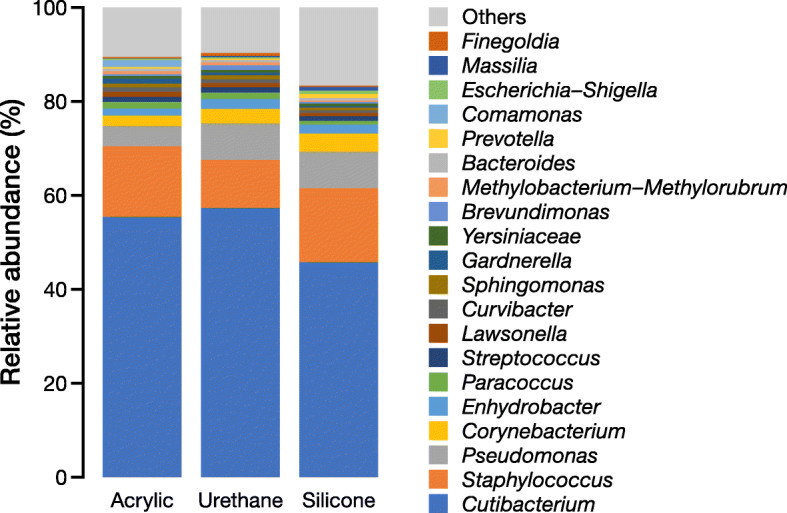
Average relative abundance of the top 20 skin microbiota collected by the three different film dressings (acrylic, urethane, and silicone adhesives)

The bacterial composition obtained from the three different film dressings is summarized in Fig. 2 (with Additional file 1: Figure S1 for each participant). All three dressings obtained the same top five genera (*Cutibacterium* species [spp.], *Staphylococcus* spp., *Pseudomonas* spp., *Corynebacterium* spp., and *Enhydrobacter* spp.), all of which have been reported as skin commensals [22, 23]. However, the relative abundances of these bacteria were apparently different between the silicone adhesive and the other two adhesives.

**Fig. 3:**
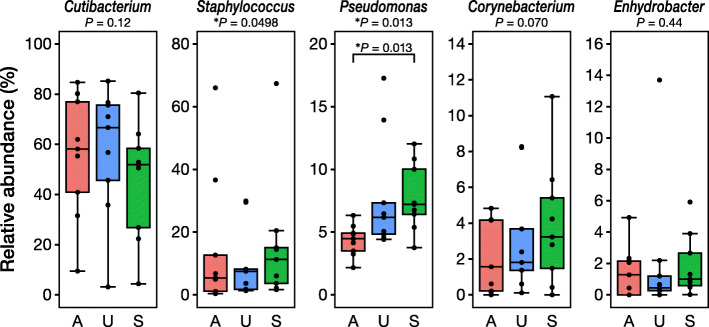
Relative abundance of the top five skin microbiota collected by the three different film dressings. The *P* values below the name of the bacteria are the results of the Friedman test, whereas the *P* value inside each plot was calculated by the Nemenyi *post-hoc* pairwise test. ^*^P < 0.05. A, acrylic adhesive; U, urethane adhesive; S, silicone adhesive. For details, please refer to Table 1

Detailed comparisons of the top five genera are shown in Fig. 3, and a top 20 comparison is shown in Table 1. Of the top five genera, *Pseudomonas* spp. showed a significant difference between acrylic and silicone adhesives (*P* = 0.013). The other top five bacteria did not show any significant differences between the three film dressings, although the silicone adhesive yielded slightly different values compared with the other two adhesives. The results of the amplicon sequence variants (ASVs)-level comparison, in which ASVs that showed more than 0.1% of average relative abundance are listed, are shown in Additional file 2: Table S1. Similar to the genus-level comparison, the silicone adhesive showed significantly different value in one ASV (9th rank ASV).

**Table 1:** Relative abundances and results of statistical evaluation of the top 20 genera obtained using tape stripping with three different types of adhesive (acrylic, urethane, and silicone) to evaluate skin microbiota

	Relative abundance (%)			Friedman test ***P*** value	***P*** value of Nemenyi ***post-hoc*** test^a^		
Genus	Acrylic	Urethane	Silicone		A–U	A–S	U–S
*Cutibacterium*	58.2 (40.9–77.0)	66.7 (45.7–75.6)	51.9 (26.8–58.4)	0.12			
*Staphylococcus*	5.4 (1.2–12.7)	7.5 (1.8–8.2)	11.3 (3.7–15.0)	0.0498^*^	1.0	0.086	0.086
*Pseudomonas*	4.5 (3.5–4.9)	6.2 (4.8–7.3)	7.2 (6.4–10.0)	0.013^*^	0.086	0.013^*^	0.76
*Corynebacterium*	1.6 (0.2–4.2)	1.8 (1.4–3.7)	3.2 (1.5–5.4)	0.070			
*Enhydrobacter*	1.3 (0.0–2.1)	0.4 (0.3–1.2)	1.0 (0.6–2.7)	0.44			
*Paracoccus*	0.8 (0.1–1.8)	0.4 (0.0–1.1)	0.8 (0.2–1.2)	0.14			
*Streptococcus*	0.0 (0.0–0.4)	0.3 (0.0–0.6)	0.8 (0.3–1.5)	0.069			
*Lawsonella*	0.7 (0.4–2.4)	0.5 (0.3–1.2)	0.6 (0.3–0.8)	0.29			
*Curvibacter*	0.5 (0.1–0.7)	0.4 (0.2–1.4)	0.5 (0.3–0.9)	0.89			
*Sphingomonas*	0.6 (0.2–1.0)	0.3 (0.3–1.7)	0.5 (0.0–0.9)	0.92			
*Gardnerella*	0.0 (0.0–0.0)	0.0 (0.0–0.0)	0.0 (0.0–0.3)	0.76			
*Yersiniaceae*	0.4 (0.4–0.5)	0.6 (0.3–0.9)	0.6 (0.3–0.8)	0.46			
*Brevundimonas*	0.2 (0.0–0.6)	0.0 (0.0–0.3)	0.2 (0.0–0.3)	0.38			
*Methylobacterium–Methylorubrum*	0.5 (0.3–1.0)	0.1 (0.0–0.8)	0.3 (0.0–0.4)	0.24			
*Bacteroides*	0.0 (0.0–0.2)	0.0 (0.0–0.5)	0.1 (0.0–0.9)	0.23			
*Prevotella*	0.0 (0.0–0.2)	0.1 (0.0–0.7)	0.1 (0.0–0.8)	0.65			
*Comamonas*	0.0 (0.0–0.0)	0.0 (0.0–0.0)	0.0 (0.0–0.0)	0.37			
*Escherichia–Shigella*	0.0 (0.0–0.2)	0.2 (0.0–0.4)	0.3 (0.0–1.3)	0.38			
*Massilia*	0.1 (0.0–0.3)	0.0 (0.0–0.4)	0.3 (0.0–0.3)	0.89			
*Finegoldia*	0.1 (0.0–0.5)	0.0 (0.0–1.1)	0.0 (0.0–0.3)	0.83			

Data are expressed as the median (interquartile range)

*A* Acrylic adhesive, *U* Urethane adhesive, *S* Silicone adhesive

^*^P < 0.05

^a^Adjusted *P* value by the Holm method; only evaluated when the Friedman test was significant

### Beta diversity

**Fig. 4:**
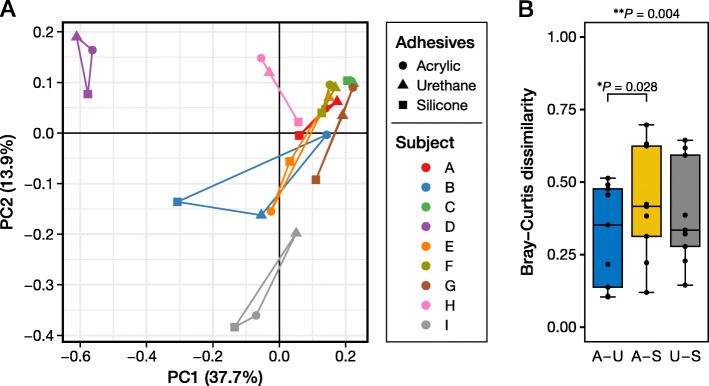
Beta diversity plot based on Bray–Curtis dissimilarity (**a**) and pairwise comparison of Bray–Curtis dissimilarity for the three different dressings (**b**). **a** Each different color denotes each participant. The plots from the same participant are connected by lines. **b** The *P* value outside the box is the result of the Friedman test, whereas the *P* value inside the box is calculated by the Nemenyi *post-hoc* pairwise test. ^*^P < 0.05, ^**^P < 0.01. PC, principal coordinate; A, acrylic adhesive; U, urethane adhesive; S, silicone adhesive. The blue box in (**b**), A vs. U; the yellow box, A vs. S; the gray box, U vs. S

We performed a principal coordinate analysis plot based on Bray–Curtis dissimilarity (Fig. 4a). There were no statistically significant differences in the pattern of beta diversity, as evaluated by the pairwise permutational multivariate analysis of variance (PERMANOVA) analysis (Table 2). The Bray–Curtis dissimilarity itself showed a significantly higher value between the acrylic- and silicone-adhesive film dressings than between the acrylic- and urethane-adhesive film dressings (*P* = 0.028; Fig. 4b).

**Table 2:** Results of PERMANOVA for beta diversity (Bray–Curtis dissimilarity)

	*P* value	*q* value^a^
Overall	0.77	–
Acrylic – Urethane	0.96	0.96
Acrylic – Silicone	0.41	0.91
Urethane – Silicone	0.61	0.91

^a^Adjusted *P* value by means of the Benjamini–Hochberg false discovery rate control

### Alpha diversity

**Fig. 5:**
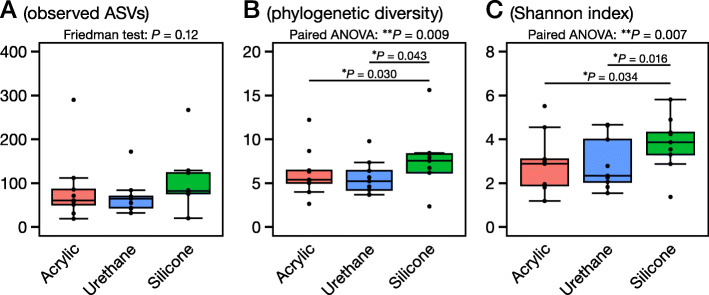
Alpha diversity indices: the number of observed amplicon sequence variants (ASVs) (**a**), phylogenetic diversity (**b**), and Shannon index (**c**). The *P* values outside the box are the results from the Friedman test or one-way analysis of variance (ANOVA) of repeated measures, whereas the *P* values inside each box were calculated by the Nemenyi *post-hoc* pairwise test or Welch’s pairwise *t*-test with Holm adjustment

The alpha diversity indices (observed ASVs, phylogenetic diversity, and Shannon index) are shown in Fig. 5. The silicone-adhesive film dressing showed significantly higher alpha diversity indices than the acrylic or urethane adhesive film dressing, except for observed ASVs.

## Discussion

We tested film dressings with three different adhesives (acrylic, urethane, and silicone) as a means of collecting skin microbiota samples. We found that acrylic and urethane adhesives obtained similar results, and the use of silicone-adhesive film dressing led to biased outcomes for amplicon-based skin microbiome analyses.

It is crucial to collect the largest possible amount of skin microbiota (i.e., 16S rRNA genes) to obtain reliable skin microbiome data [24]. To assess the differences in the yield of skin microbiota, we first determined the copy number of 16S rRNA genes obtained from the three film dressings, and then we compared the results using a previously-validated dressing with acrylic adhesive as a benchmark [12]. As expected, the film dressing with acrylic adhesive yielded the highest number of 16S rRNA genes, followed by the film dressing with urethane adhesive and then the film dressing with silicone adhesive (Fig. 1). Film dressings with silicone adhesive were developed to reduce the attachment force to the skin and were reported to require less removing force and to reduce skin damage and discomfort [16, 17, 21, 25, 26]. The significantly lower efficiency of silicone-adhesive dressings in collecting skin microbiota could be due to the reduced power of attachment to the skin. Because the use of urethane adhesive for film dressings is a relatively new concept, only a limited number of film dressings with urethane adhesive were commercially available. The urethane adhesive product that we used employed a urethane gel as an adhesive with a view to high adhesiveness and low irritation [20]. The number of 16S rRNA genes obtained by the urethane adhesive fell between the amount obtained by the acrylic and silicone adhesives and was not significantly less than the acrylic adhesive (Fig. 1). Bender et al. reported that microbial results could be biased if the input copy number of 16S rRNA genes was low and suggested that at least 100 copies were required for NGS analysis [24]. From our study, when assuming that 1 μL of the DNA solution is used as a template for NGS, no dressings with acrylic adhesive, 1/9 dressings with urethane adhesive, and 5/9 dressings with silicone adhesive did not reach the threshold (100 copies, [24]) (Fig. 1). Therefore, it is expected that dressings with acrylic or urethane adhesive would be the first choice for amplicon-based skin microbiome studies, prioritized according to yield or safety, respectively.

Next, we assessed the microbiome results obtained from the three film dressings. All three film dressings had the same top five skin microbiota (Figs. 2 and 3, and Table 1), namely, *Cutibacterium* spp., *Staphylococcus* spp., *Pseudomonas* spp., *Corynebacterium* spp., and *Enhydrobacter* spp. However, the dressing with silicone adhesive showed significantly different results in the relative abundance of *Pseudomonas* spp., and less, albeit not significant, *Cutibacterium* spp. (Figs. 2 and 3, and Table 1). The ASV-level analysis also showed simiar results in which the dressing with silicone adhesive yielded different results in comparison with the dressing with acrylic or urethane adhesive (Additional file 2: Table S1). Additionally, the Bray–Curtis dissimilarity was significantly higher between the acrylic- and silicone-adhesive film dressings than between the acrylic and urethane film dressings (Fig. 4b), although there were no significant differences in the principal coordinate analysis distribution as a whole (Fig. 4a). Finally, the dressing with silicone adhesive showed significantly higher alpha diversity indices (phylogenetic diversity and Shannon index) than the other two film dressings (Fig. 5). Thus, we presumed that the film dressing with silicone adhesive leads to subtly different results compared with the other two adhesive film dressings. This can be explained by differences in the amount of template and PCR cycles. In this study, for the samples with a smaller 16S rRNA gene copy number (especially those derived from the film dressing with silicone adhesive), the number of PCR cycles was increased by 1–4 cycles to achieve a sufficient amount of amplified product (Additional file 3: Table S2). Brooks et al. reported that the number of PCR cycles could slightly affect the results of the 16S rRNA gene sequence data [27]. Given the situation where samples with an insufficient amount of template (Fig. 1) were amplified to achieve sufficient product for NGS analysis, it is possible that the differences in the cycling conditions might have affected the results. However, Sze and Schloss mentioned that KAPA HiFi HS enzyme, which was used in this study, gave the most stable results, irrespective of a higher number of PCR cycles, among the PCR enzymes tested [28]. Furthermore, the use of KAPA HiFi HS enzyme was reported to lead to a lower Shannon index when the PCR cycles were increased [28], which contradicts the findings of this study. Therefore, there may be another explanation for why the dressing with silicone adhesive yielded slightly different results.

Our study had several limitations. First, we only tested one position (back skin), as in our previous study [12], and a different position might give different results, as the skin microbiome is diverse across body sites [29]. Second, we did not test the three film dressings on fragile skin (e.g., older people), so we could not directly prove the applicability and safety of the film dressing with a urethane adhesive to those who have fragile skin. Nonetheless, we have already utilized the urethane-adhesive film dressing for skin microbiome study on older people with fragile skin (age range, 71–100 years old), and there were no adverse events [23]; therefore, the dressing with urethane adhesive can be considered safe as such for very old people. Third, the number of participants might be small for comparison. In this study, we have recruited nine participants as with our previous study [12] and the studies of other groups [22, 30–33]. The smaller number of samples leads to less statistical power for significance tests; therefore, the non-significant difference in the microbial composition between acrylic and urethane dressings found in this study could also be explained by the small number of samples. However, at least, silicone adhesive would not be recommended for skin microbiome studies, as it showed significantly less microbial yield and different microbial composition even under small number of samples. That being said, it would be advised that the sampling method should be fixed (i.e., acrylic *or* urethane) throughout a single study. Finally, more detailed comparison by whole genome sequencing with species- or strain-level analysis would give different results when compared to amplicon-based metagenomics. Further studies with whole genome metagenomics are warranted.

## Conclusion

This study showed that film dressings with acrylic or urethane adhesive should be chosen when used to obtain skin samples for amplicon-based microbiome studies. The choice of acrylic or urethane adhesive can be based on what requires prioritization: acrylic adhesive for the yield of skin microbiota or urethane adhesive for skin safety, such as when sampling from older people. A film dressing with silicone adhesive was unsuitable for the purpose of skin microbiome studies, yielding measurably less skin microbiota and subsequent biased results.

## Methods

### Ethical approval

This study was approved by the Medical Ethics Committee of Kanazawa University, Kanazawa, Japan (approval number 803). The researchers followed the Declaration of Helsinki and the Microorganism Safety Management Regulations of Kanazawa University. Bacterial samples were processed in a level 2 biosafety laboratory.

### Participants

We recruited nine healthy young participants (four females, age 21–22 years old; five males, age 20–21 years old). Written informed consent was obtained from all participants prior to inclusion. No participants had skin disorders or any systemic diseases. Additionally, none of the participants reported the use of topical or systemic antibiotics within 2 weeks prior to study inclusion. The participants were requested to not use creams or lotions on the site of sample collection (back skin) within 24 h of the day of sampling and to not take a bath or shower after midnight on the day before sampling. All the participant were confirmed to have complied with the requests.

### Film dressings

We selected three types of film dressings with different adhesives for tape stripping: (1) a film dressing with acrylic adhesive (Tegaderm™ Transparent Film Dressing, 1622 W; 3 M Company, MN, USA), (2) a film dressing with urethane adhesive (Cathereeplus™, CPS0405; Nichiban Co., Ltd., Tokyo, Japan), and (3) a film dressing with silicone adhesive (Mepitel Film, 296,170; Mölnlycke Health Care, Gothenburg, Sweden). The size of each film dressing was ~ 20 cm^2^ (acrylic, 4.4 × 4.4 cm, 19.4 cm^2^; urethane, 4 × 5 cm, 20 cm^2^; and silicone, 6 × 7 cm, which was cut in half to 6 × 3.5 cm, 21 cm^2^).

### Tape stripping

Tape stripping was performed with the three different film dressings. First, the participant’s back was inspected to check that there were no apparent skin diseases (e.g., rash, wound, or sores). The position (back skin) was chosen as in our previous study [12]. Then, three neighboring areas were designated, and each film dressing was applied randomly to each designated area (Additional file 4: Figure S2). All the film dressings were pressed once using a sterile roller (MSR0001; Bio-Rad Laboratories, Inc., CA, USA) with constant pressure to ensure attachment to the skin. After 1 min, the film dressings were removed from the skin using sterile round-tip tweezers and placed in separate sterile plastic containers. The film dressings were stored at − 80 °C until DNA extraction.

### DNA extraction

Bacterial DNA was extracted from the film dressings as described previously [12, 34]. Briefly, using a safety cabinet, each film dressing was minced by sterile scissors, followed by chemical (1.2% Triton-X 100) and enzymatical (lysozyme and lysostaphin) disruption of the bacterial cell walls. The resultant solution was then cleaned-up using a QIAamp DNA Mini Kit (QIAGEN, Venlo, The Netherlands) according to the manufacturer’s instructions. Each extracted bacterial DNA sample was recovered in 100 μL of AE buffer (10 mM Tris-HCl, 0.5 mM ethylenediaminetetraacetic acid, pH 9.0). The DNA solution was stored at − 20 °C until analysis.

### Library preparation for NGS

Quantitative polymerase chain reaction (qPCR) for 16S rRNA gene quantification was performed on the extracted bacterial DNA, as described elsewhere [12], with THUNDERBIRD® SYBR® qPCR Mix (Toyobo Co., Ltd., Osaka, Japan) and 16S rRNA primers (forward: 5′-ACTGAGAYACGGYCCA-3′ and reverse: 5′-CTGCTGGCACGDAGTTAGCC-3′) on an AriaMX Real-Time PCR System (Agilent Technologies, Inc., CA, USA). Then, equal concentrations of the resultant amplified DNA solutions containing the 16S rRNA gene were used to amplify the V3–V4 hypervariable region, to better capture the microbial composition of the skin [30], with KAPA HiFi HS ReadyMix (F. Hoffmann-La Roche, Ltd., Basel, Switzerland) and primers with MiSeq-compatible overhang sequences (forward: 5′-TCGTCGGCAGCGTCAGATGTGTATAAGAGACAGCCTACGGGNGGCWGCAG-3′ and reverse: 5′-GTCTCGTGGGCTCGGAGATGTGTATAAGAGACAGGACTACHVGGGTATCTAAKCC-3′). After cleaning up the polymerase chain reaction (PCR) products using AMPure XP magnetic beads (Beckman Coulter Inc., CA, USA), indexing PCR was performed for all samples using a Nextera XT Index Kit v2 (Illumina Inc., CA, USA) followed by AMPure XP cleaning. The concentration of each product was measured by means of a Qubit dsDNA HS Assay Kit (Thermo Fisher Scientific, MA, USA), and equimolar amounts of the products were mixed to create the final library solution.

### NGS

The library solution was spiked with 15% PhiX Control v3 (Illumina Inc.) and loaded in a flow cell of a MiSeq Reagent Kit v3 (600 cycles; Illumina Inc.) on a MiSeq system (Illumina Inc.). NGS was performed according to the manufacturer’s instructions.

### Bioinformatics analyses

All the microbiome analyses were performed using QIIME 2 software (version 2019.10) [35] and the SILVA rRNA database (release 138) [36]. The raw fastq sequence files were first filtered, and chimeras were eliminated using the DADA2 plugin [37] with default settings. The taxonomic assignment was then performed using the classify-sklearn method with the trained classifier created by the SILVA database and the fit-classifier-naive-bayes method in the q2-feature-classifier plugin [38]. Alpha diversity indices (the number of observed ASVs, Faith’s phylogenetic diversity, and Shannon index) and beta diversity (based on Bray–Curtis dissimilarity [39]) were calculated by the core-metrics-phylogenetic pipeline of the q2-diversity plugin with rarefaction at a depth of 12,627 sequences, the minimum read number among all the samples. The rarefaction curves of all samples are shown in Additional file 5: Figure S3.

### Statistics

The data were expressed as averages for the cumulative bar charts of relative bacterial abundance or as medians with interquartile range (IQR) and median − 1.5 × IQR to median + 1.5 × IQR whiskers for the other parameters. The 16S rRNA gene copy number, relative abundances of representative bacterial genera, alpha diversity indices, and dissimilarity index for beta diversity among the three different film dressings were evaluated by the Friedman test followed by the Nemenyi *post-hoc* multiple comparison test using the PMCMRplus package [40] or one-way analysis of variance (ANOVA) of repeated measures followed by pairwise Welch’s *t*-test with *P* value adjustment by the Holm method using the car package [41], according to data distribution. The difference in beta diversity among the three film dressings was statistically evaluated by PERMANOVA tests followed by *P* value adjustment with Benjamini–Hochberg false discovery rate (FDR) control using beta-group-significance visualizer of QIIME 2. A *P* value or *q* value (adjusted *P* value by FDR control) < 0.05 was considered statistically significant. For statistical evaluation, R software (version 3.6.2) [42] and the QIIME 2 built-in analyzer were used.

### Data availability

All raw fastq sequences are available from the DNA Data Bank of Japan (accession no. DRA009701).

## Supplementary Information

**Additional file 1: Figure S1.** Relative abundance of the top 20 genera of skin microbiota for each participant. A, acrylic adhesive; U, urethane adhesive; S, silicone adhesive.

**Additional file 2: Table S1.** Amplicon sequence variant (ASV)-level comparison between three different adhesives. ASVs that shared more than 0.1% on average are listed.

**Additional file 3: Table S2.** Cycle numbers in the first PCR.

**Additional file 4: Figure S2.** Position of sample collection.

**Additional file 5: Figure S3.** Rarefaction curves for each alpha diversity index. ASV, amplicon sequence variants. Vertical bars denote 12,627 reads, the minimum read number among all the samples.

## Data Availability

The datasets generated and/or analysed during the current study are available in the DNA Data Bank of Japan (accession no. DRA009701). https://ddbj.nig.ac.jp/DRASearch/submission?acc=DRA009701
