# Supplementary material for: Amplicon-based skin microbiome profiles collected by tape stripping with different adhesive film dressings: a comparative study
Source: BMC Microbiol. 2021 Feb 18;21:54. doi: 10.1186/s12866-021-02122-4 (PMC7891171; doi:10.1186/s12866-021-02122-4)
Supplement: Supplementary file 1 — Additional file 1: Figure S1. Relative abundance of the top 20 genera of skin microbiota for each participant. A, acrylic adhesive; U, urethane adhesive; S, silicone adhesive. [file 12866_2021_2122_MOESM1_ESM.pdf]

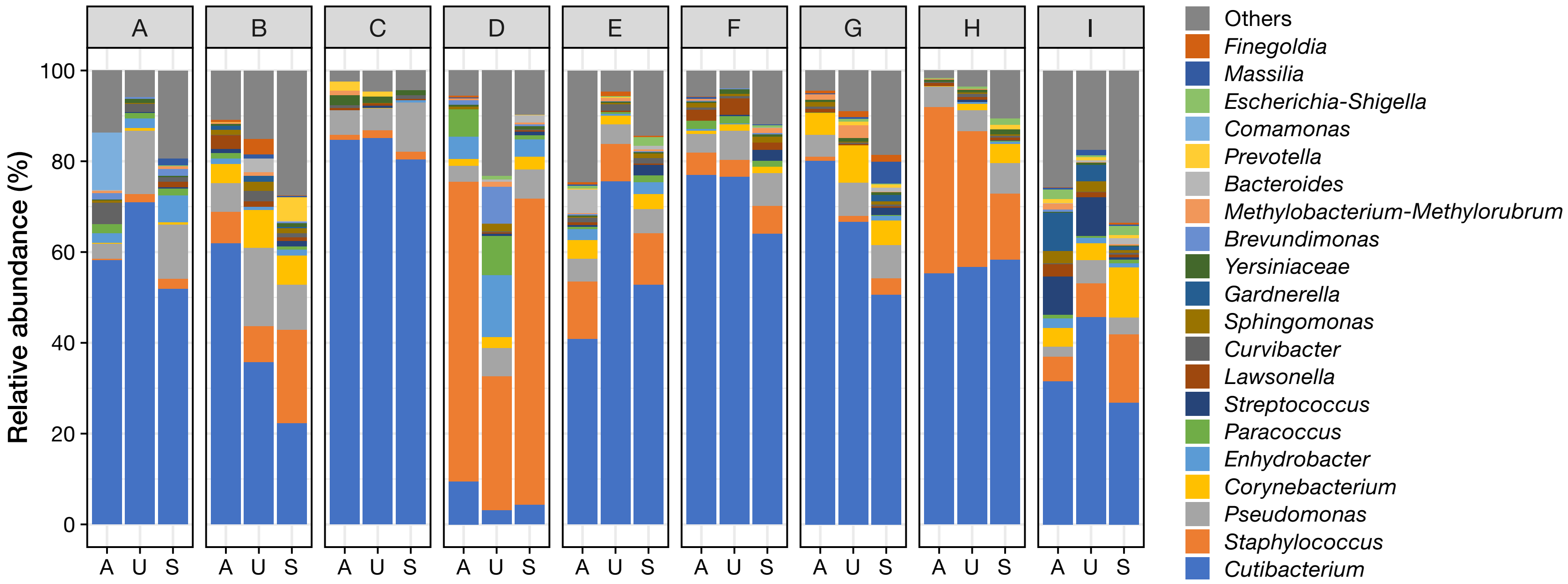

Figure S1. Relative abundance of the top 20 genera of skin microbiota for each participant.

A, acrylic adhesive; U, urethane adhesive; S, silicone adhesive.
