# Supplementary material for: Amplicon-based skin microbiome profiles collected by tape stripping with different adhesive film dressings: a comparative study
Source: BMC Microbiol. 2021 Feb 18;21:54. doi: 10.1186/s12866-021-02122-4 (PMC7891171; doi:10.1186/s12866-021-02122-4)
Supplement: Supplementary file 3 — Additional file 3: Table S2. Cycle numbers in the first PCR. [file 12866_2021_2122_MOESM3_ESM.pdf]

Table S2. Cycle numbers in the first PCR.

Sample ID	Acrylic	Urethane	Silicone
A	30	31	32
B	30	33	34
C	30	30	30
D	30	31	33
E	30	30	32
F	30	30	30
G	30	30	32
H	30	30	30
I	30	30	31
