# Supplementary figures and images for: Amplicon-based skin microbiome profiles collected by tape stripping with different adhesive film dressings: a comparative study
Source: BMC Microbiol. 2021 Feb 18;21:54. doi: 10.1186/s12866-021-02122-4 (PMC7891171; doi:10.1186/s12866-021-02122-4)

**Back view**

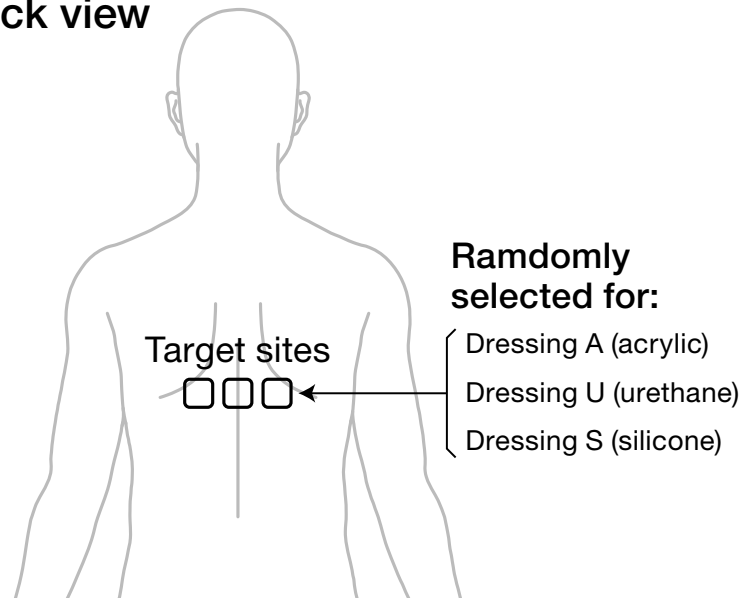

**Figure S2. Position of sample collection.**

Supplement: Supplementary file 4 — Additional file 4: Figure S2. Position of sample collection. [file 12866_2021_2122_MOESM4_ESM.pdf]
