# Supplementary material for: Amplicon-based skin microbiome profiles collected by tape stripping with different adhesive film dressings: a comparative study
Source: BMC Microbiol. 2021 Feb 18;21:54. doi: 10.1186/s12866-021-02122-4 (PMC7891171; doi:10.1186/s12866-021-02122-4)
Supplement: Supplementary file 5 — Additional file 5: Figure S3. Rarefaction curves for each alpha diversity index. ASV, amplicon sequence variants. Vertical bars denote 12,627 reads, the minimum read number among all the samples. [file 12866_2021_2122_MOESM5_ESM.pdf]

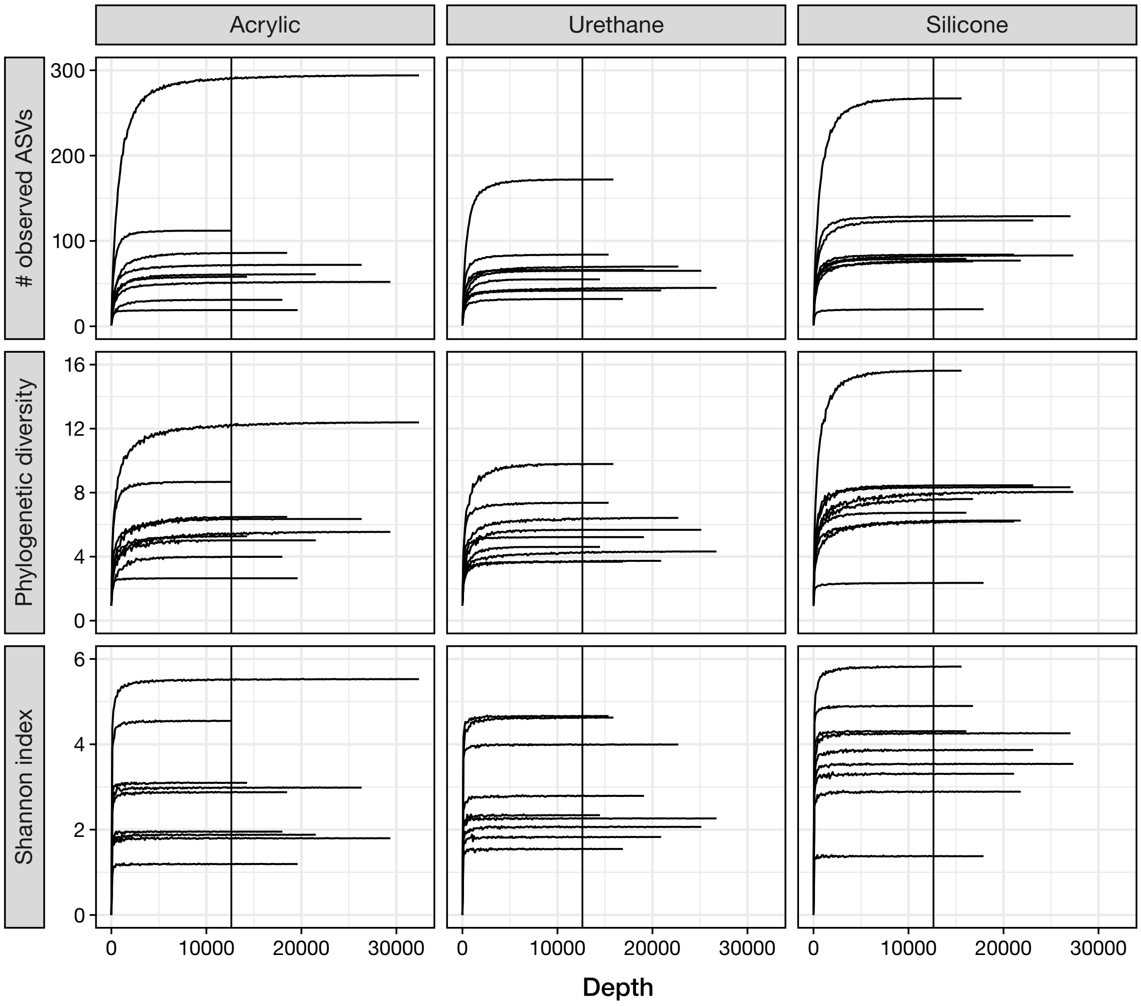

Figure S3. Rarefaction curves for each alpha diversity index.

ASV, amplicon sequence variants. Vertical bars denote 12627 reads, the minimum read number among all the samples.
